# Development of a Stepping Force Analgesic Meter for a Rat Arthritic Model

**DOI:** 10.3390/s110505058

**Published:** 2011-05-05

**Authors:** Mun Fei Yam, Lip Yee Por, Kok Khiang Peh, Mariam Ahmad, Mohd. Zaini Asmawi, Lee Fung Ang, Delina Beh Mei Yin, Sim Ying Ong, Muthanna Fawzy Abdulkarim, Ghassan Zuhair Abdullah, Ibrahim Muhammad Salman, Omar Ziad Ameer, Elsnoussi Ali Hussin Mohamed, Mohd Akmal Hashim, Elham Farsi, Sook Yee Hor

**Affiliations:** 1 School of Pharmaceutical Sciences, Universiti Sains Malaysia, 11800, Penang, Malaysia; E-Mails: kkpeh@usm.my (K.K.P.); mariam@usm.my (M.A.); amzaini@usm.my (M.Z.A.); ang.leef@gmail.com (L.F.A.); muthana_albuldawy@yahoo.com (M.F.A.); ghassanalyasiri@yahoo.com (G.Z.A.); ibraheem_muhammed@yahoo.com (I.M.S.); omar_3m@yahoo.com (O.Z.A.); aliali692002@yahoo.com (E.A.H.M.); mal_roteiro85@yahoo.com.my (M.A.H.); elhamfarsi@gmail.com, (E.F.); sookyee4321@yahoo.com (S.Y.H.); 2 Faculty of Medicine and Health Sciences, Universiti Putra Malaysia, 43400, Serdang, Malaysia; 3 Faculty of Computer Science and Information Technology, University of Malaya, 50603, Kuala Lumpur, Malaysia; E-Mails: porlip@um.edu.my (L.Y.P.); tomatoying@yahoo.com (S.Y.O.); 4 Malaysian Institute of Information Technology, Universiti Kuala Lumpur, 50250, Kuala Lumpur, Malaysia; E-Mail: delina@miit.unikl.edu.my (D.B.M.Y.)

**Keywords:** arthritis, analgesimeter, nociception, stepping force, load cells

## Abstract

Behavioural assessment of experimental pain is an essential method for analysing and measuring pain levels. Rodent models, which are widely used in behavioural tests, are often subject to external forces and stressful manipulations that cause variability of the parameters measured during the experiment. Therefore, these parameters may be inappropriate as indicators of pain. In this article, a stepping-force analgesimeter was designed to investigate the variations in the stepping force of rats in response to pain induction. The proposed apparatus incorporates new features, namely an infrared charge-coupled device (CCD) camera and a data acquisition system. The camera was able to capture the locomotion of the rats and synchronise the stepping force concurrently so that each step could be identified. Inter-day and intra-day precision and accuracy of each channel (there were a total of eight channels in the analgesimeter and each channel was connected to one load cell and one amplifier) were studied using different standard load weights. The validation studies for each channel also showed convincing results whereby intra-day and inter-day precision were less than 1% and accuracy was 99.36–100.36%. Consequently, an *in vivo* test was carried out using 16 rats (eight females and eight males). The rats were allowed to randomly walk across the sensor tunnel (the area that contained eight channels) and the stepping force and locomotion were recorded. A non-expert, but from a related research domain, was asked to differentiate the peaks of the front and hind paw, respectively. The results showed that of the total movement generated by the rats, 50.27 ± 3.90% in the case of the male rats and 62.20 ± 6.12% in that of the female rats had more than two peaks, a finding which does not substantiate the assumptions made in previous studies. This study also showed that there was a need to use the video display frame to distinguish between the front and hind paws in the case of 48.80 ± 4.01% of the male rats and 66.76 ± 5.35% of the female rats. Evidently the assumption held by current researchers regarding stepping force measurement is not realistic in terms of application, and as this study has shown, the use of a video display frame is essential for the identification of the front and hind paws through the peak signals.

## 1. Introduction

Arthritis, a degenerative and debilitating disease, is associated with chronic pain of the joints, which can impair the ability to work and also lead to severe psychological and social problems [1]. Osteoarthritis is suffered by 15% of the world population [2], whereas rheumatoid arthritis, a chronic inflammatory illness, affects about 1% of the world’s population [3]. Rodent models of arthritis have been developed to help in elucidating the underlying pathophysiology involved in arthritis by identifying specific modulators or receptors involved in the pain process. To date, several behavioural tests for analgesic quantification using rats have been developed, which include: (i) paw withdrawal threshold and latency [4–6], (ii) withdrawal response to radiant heat [7] and (iii) arthritic rat walking on a rotating cylinder [8]. Although these tests provide valuable information about the pain mechanism and potential pharmacological therapies, they do suffer several drawbacks from a practical point of view. These tests often involve stressful manipulations of the animals [8–10], and the parameters measured in certain tests are not always suitable as indicators of pain. In order to prevent the stress factor on the rat, researchers have claimed [10] that only the use of the weight distribution model can be considered a fair measurement as it is able to provide an objective and non-evoked assessment of persistent chronic pain in animals. In 1995, the concept of measuring ground reaction force during rat locomotion was put forward by Clarke [11]. Forces and pressures exerted via fore and hind paws can be measured when the rat walks. Images from the ambulating rat were videoed via a mirror at 45 degrees using a camera capturing images at 25 frames per second. Then, the camera captured both the monitor display of load cell output together with the corresponding paw contacts with the load cell platform [11]. Each recorded video frame was digitised in a frame grabber to produce an image with 256 × 256 pixels per frame. In the study, Clarke [11] only measured the paws and limbs through the peak signal from their stepping forces. However, Clarke [11] neglected the synchronisation between the stepping force and the image where the speed of the locomotion can vary unexpectedly.

In 2001 Min *et al.* constructed a device to measure the weight load on each leg while the animal was walking through a path; the bottom of the device was equipped with strain gauge weight sensors [10]. This device helped to measure the weight load on the right hind leg. According to the researchers, decreased weight bearing in arthritic animals is one of the most commonly observed functional disabilities in such animals as well as arthritic human beings. Min *et al.* claimed that the proposed device was an effective tool for convenient measurement of arthritic pain, the advantage being that it captures a dynamic condition—the legs of a voluntarily walking rat. The rat is not restrained nor forced to maintain its static position [12]. Since the device measured weight load while the rat was walking freely, the state of the arthritic pain was reflected realistically. The limitation of this device is that no camera was installed to capture the real-time movements, thus the peak caused by the front paw could not be differentiated from that of the hind paw. Besides, the software used is proprietary and not customisable to the researcher’s requirement. For example, the stepping force and the movement of the rodent cannot be synchronised.

After reviewing relevant literature, we concluded that no research related to the development of an analgesic meter associated with a well-programmed data acquisition system for investigating the standing weight force of rats has been published. Hence, a new prototype to measure the stepping force of the rodents was proposed. The proposed apparatus was fabricated with a new feature which was equipped with a built-in infrared CCD camera integrated with the analgesimeter. The camera is able to capture the locomotion of the rats and synchronise the stepping force concurrently, so each step can be correctly identified.

## 2. Experimental Section

### 2.1. Fabrication of the Analgesic Meter

**Figure 1.**
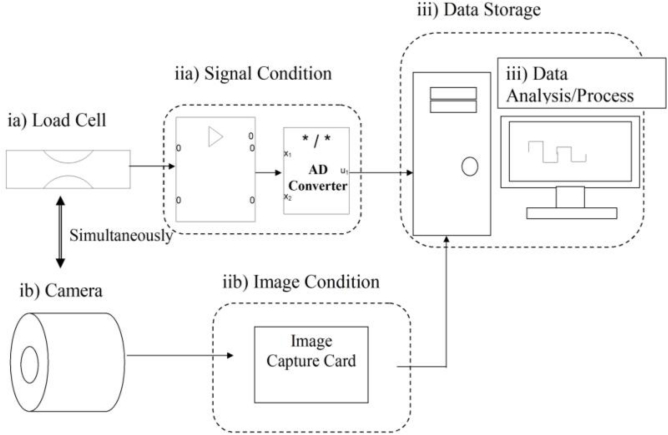
Block diagram of the analgesimeter. During the rat locomotion, the stepping forces while the animal was walking across the sensor tunnel were measured by a load cell **(ia)** and images of the movements were captured by an infrared video camera **(ib)** simultaneously. The signals were amplified by an amplifier which was digitised by an analogue-digital converter **(iia)** and the images were processed by an image capture card **(iib)** before being stored in a hard disk **(iii)**.

The design of the proposed system consists of an 8-channel analgesimeter and a data acquisition system. There are four main components in the analgesimeter: (i) apparatus, (ii) amplifiers station, (iii) video camera box and (iv) computer. Figure 1 shows the block diagram of the analgesimeter.

The apparatus was composed of a starting box, a path and an arrival box. The floor of the path consisted of eight transparent Perspex plates (width × length: 5 cm × 7 cm) attached to load cells (strain gauge type, working range 0–600 g, DA cell, Korea). The sidewalls of the path were built with two L-shaped and a rectangular (cover) black Perspex plates. The other parts of the apparatus were made of aluminium.

The output of each load cell was fed to an analogue amplifier (AM 100) (DA cell, Korea) for amplification to form a channel. The amplified signal was conducted to a personal computer via an LCPI 9112 analogue-digital converter (Adlink Tech. Inc., Taiwan). All the amplifiers were supplied with 240 V and the current was filtered (by a Cosel MAP-16-472-D noise filter, Cosel, Japan) before being supplied to each amplifier. A bakelite box was designed to host a Huper H4MR type IR CCD camera (Huper, USA). The camera was connected to a Picolo PCI card (Euresys, USA) to transfer the captured images from the CCD camera to a personal computer. The computer was used to gather data acquired by the analgesimeter.

**Figure 2.**
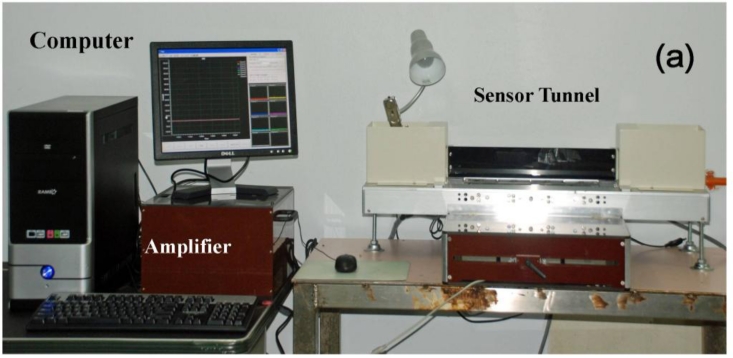

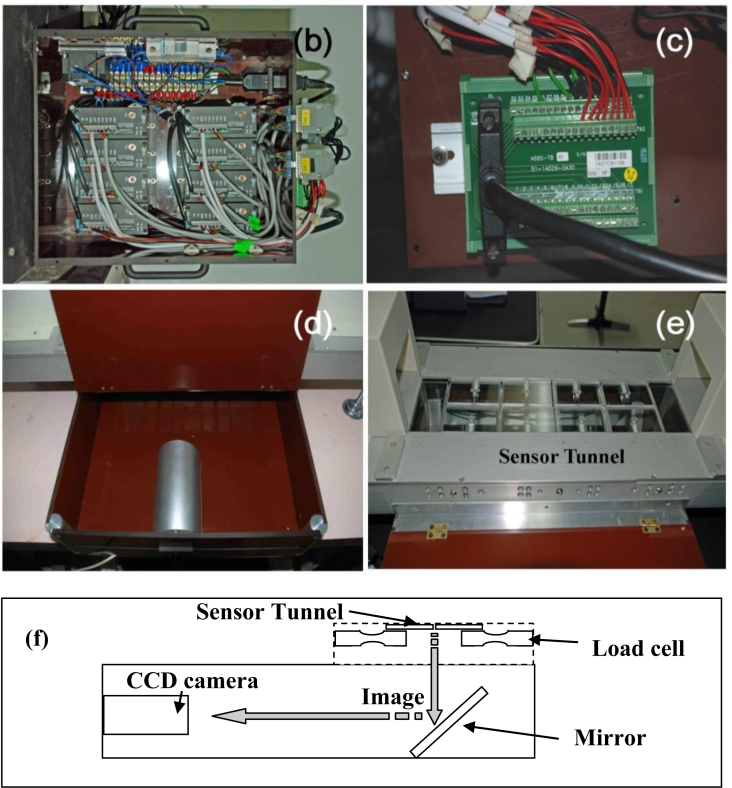
Development of the analgesimeter. **(a)** Analgesimeter. **(b)** Amplifier box. **(c)** A/D converter card. **(d)** Camera box equipped with a CCD. **(e)** Sensor tunnel (containing eight channels and each channel consists of one load cell which is connected to an amplifier). **(f)** CCD installation.

Figure 2 shows the layout of the analgesimeter. Initially, a test was carried out by placing the rat in the starting box.

While the rodent was walking through the sensor tunnel (consisting of eight channels; four on the left side and four on the right side), the weight load on a given leg of the rat could be sensed by load cells. This signal was then amplified by the amplifier in the amplifier box before being led to a computer by an analogue-digital converter. A CCD camera was also installed to capture the image reflected from the mirror in the camera box. The signals produced from the load cells and images captured by CCD camera were sequenced concurrently and stored in the personal computer using special data acquisition software. The sidewalls of the path in the apparatus were designed with two movable Perspex L-shaped plates so that the width of the path could be adjusted according to the body size of the animals.

### 2.2. Data Acquisition System

**Figure 3.**
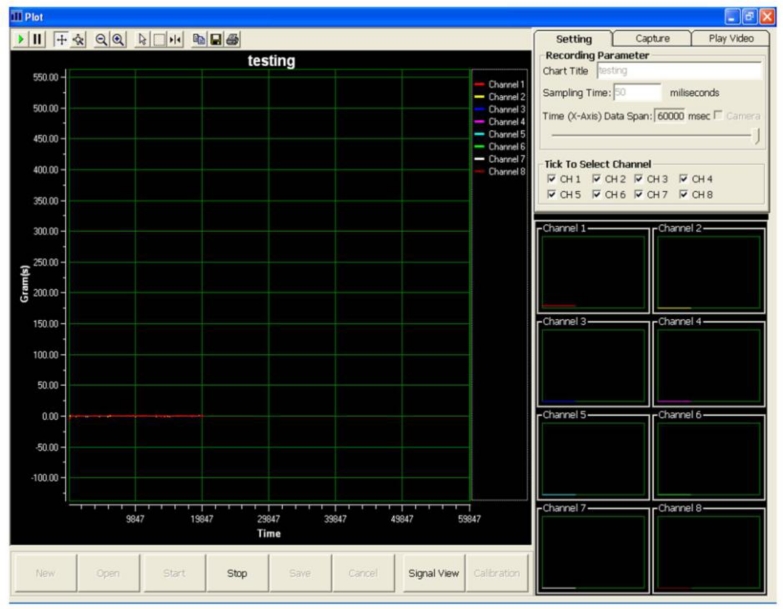
Graphical user interface of the data acquisition system.

Visual Basic 6 was used to program the data acquisition software. A graphical user interface for the data acquisition system was deployed, as shown in Figure 3. The layout of the data acquisition system consists of a real-time video display frame, eight channels signal monitoring frame and a real-time signal display frame.

**Figure 4.**
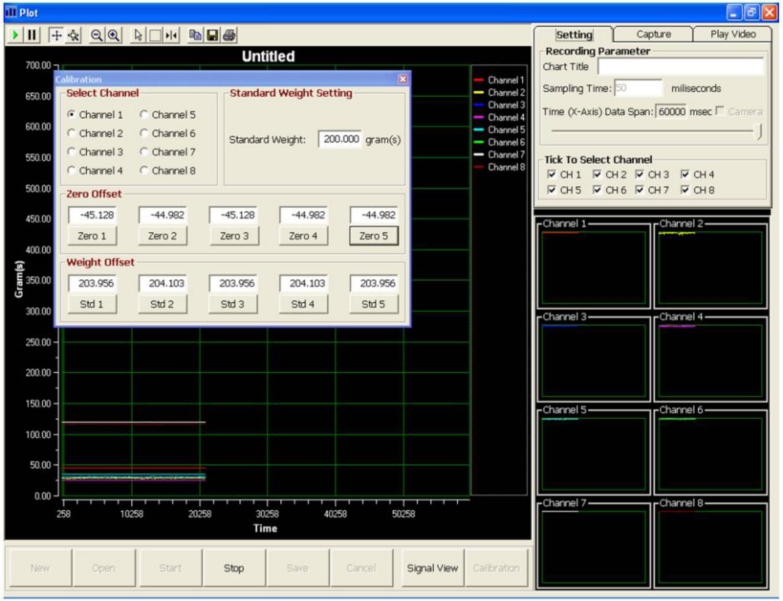
New calibration with ‘OFFSET’ and Y = mX + C linear equation methods.

To achieve a more precise and accurate measurement, a simple calibration method (Figure 4) which utilised the linear regression equation Y = mX + C [13], where:

Y = signal after interpretation (gram)
X = raw signal from amplifier (voltage)
m = slop or gradient of calibration curve
C = the meeting point of calibration curve on the y-axis

### 2.3. Validation of the Analgesimeter

#### 2.3.1. Precision

Both intra-day and inter-day precision of the analgesimeter were measured by calculating the relative standard (RSD) of the measurements. Intra-day precision and inter-day precision of each channel of the analgesimeter were studied by measuring the weights of standard loads (*i.e.*, 2, 5, 10, 20, 50, 100 and 200 g) 10 times in a single day and for six days. The RSD was calculated using the following equation:

$$\text{RSD}(\%)=\frac{\text{standard}\;\;\text{deviation}}{\text{average}}\times 100\%$$

#### 2.3.2. Accuracy

Each channel of the analgesimeter was equipped with standard load (*i.e.*, 2, 5, 10, 20, 50, 100 and 200 g) and the accuracy of each channel was calculated by comparing the value measured with reference weight according to the following equation:

$$\text{Accuracy}(\%)=\frac{\text{actual}\;\;\;\text{weight}}{\text{reference}\;\;\text{weight}}\times 100\%$$

### 2.4. *In Vivo* Study

The *in vivo* test was carried out by using 16 rats comprising eight females and eight males. A rat was randomly selected to be placed in the starting box and allowed to walk voluntarily in the sensor tunnel until the arrival box.

**Figure 5.**
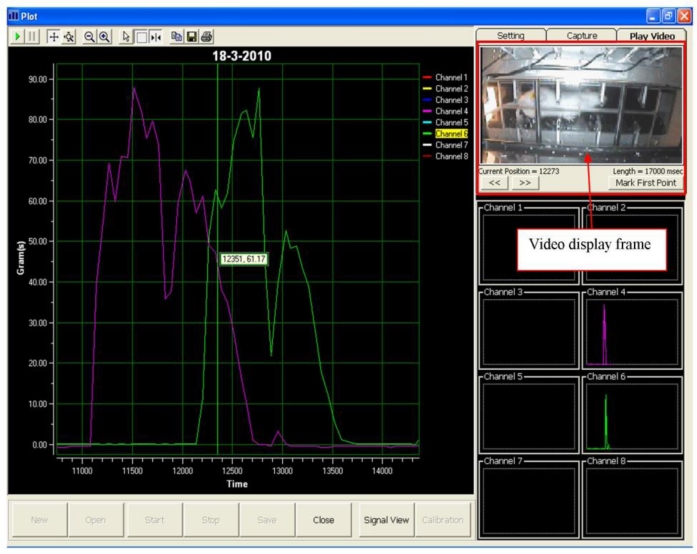
Video display frame.

Subsequently, the test was repeated at least three times a day for a total of six days. Then, the locomotion of the rat was observed to identify the peak signal of the front and hind paws. A non-expert of the related research domain was required to identify the peaks of the front and hind paw, respectively. In the event the non-expert was unable to identify the peaks, a video display frame was used to aid the non-expert in identifying the peak of the respective front and hind paw. Figure 5 shows the display frame that can be used to distinguish between the front and hind paw.

## 3. Results and Discussion

In order to prove the precision and accuracy of the measurement, the results for precision and accuracy of each channel were noted in Table 1. The means values of intra-day and inter-day precision are less than 1%. The results have also shown that the accuracy of the channels is convincing: 99.36–100.36%. From the observation, two basic rat movements can be identified, and these are smooth movement and intermittent movement. Smooth and intermittent movements are referred to the style of movement of the tested rat when walking across the sensor tunnel. Smooth movement means that the rats were no stop when walking across the sensor tunnel, the stepping generated is clear and easy to identify. Intermittent movement means that the movements of the rats were not smooth, and the rats were stopped when walking across the sensor tunnel.

**Figure 6.**
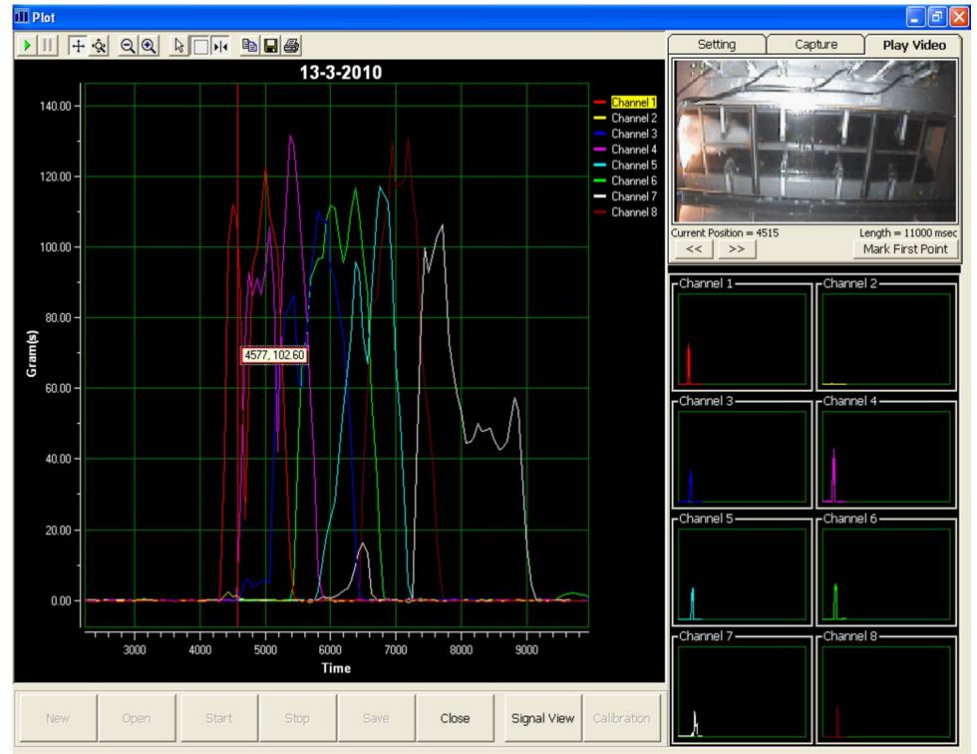
Vertical peaks show front paw and hind paw.

**Figure 7.**
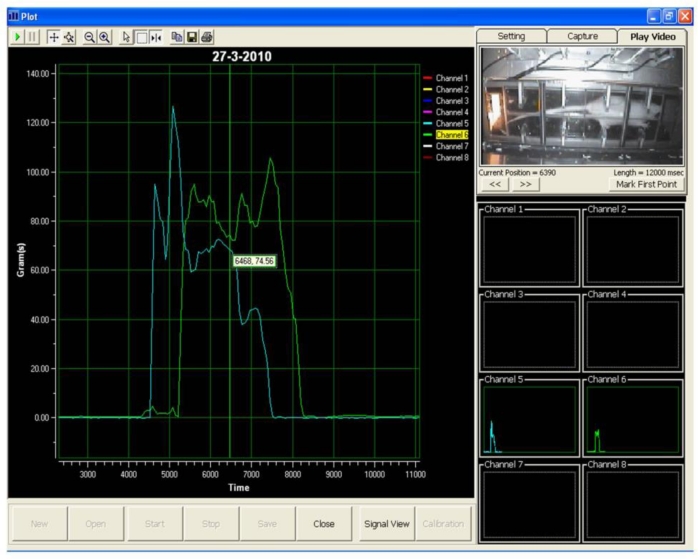
Intermittent movements of the rat.

During the test, when a rat walked voluntarily along the sensor tunnel without a halt, the manner of the rat’s movements was as indicated in Figure 6. Each and every stepping force by the front paw and hind paw is clearly shown in the eight channel signal monitoring frame. Since the tests were carried out a number of times, the rats’ movements were not always consistent and therefore, various states of peaks would appear (Figure 7). The indicator shows the state of the intermittent movement with the aid of the video display frame. For the male rats’ results, 50.27 ± 3.90% displayed intermittent movement. On the other hand, for the female rats, 62.20 ± 6.12% withheld their step on the sensor tunnel during the test. The results show that based on the observation made by the naked eye, it was difficult to conclude whether the rat made a halt or a movement.

**Figure 8.**
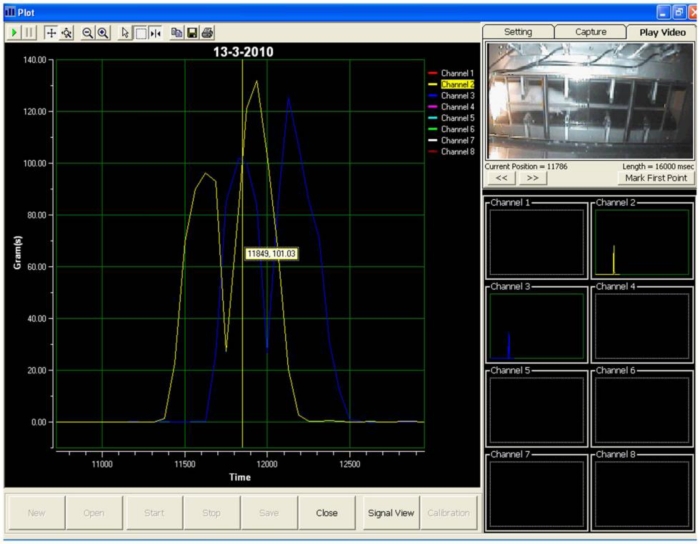
Smooth movements of the rat.

In an ideal situation, the front paw and hind paw should be clearly identifiable from the peaks, as shown in Figure 8, even without a video display frame. However, the percentage of interpretation without using the video display frame decreased approximately 51% (male rats) and 33% (female rats) by the sixth day. The percentage falls from 67% to 36% for the male rats. It can be noted that the average value of the percentage is 51.20 ± 4.01% over the six days. On the other hand, the highest and lowest percentages of interpretation without using the video display frame (female rats) are approximately 50% and 17%, respectively, and the mean of the percentage for the six days is 33.24 ± 5.35%. The percentage of remaining results requiring video display frame interpretation increased to approximately 49% and 67% for the male and female rats, respectively, on the sixth day of the test. The highest and lowest percentages of remaining results requiring video display frame to interpret the movements of the male rats are approximately 64% and 33%, respectively. The mean of the percentage is 48.80 ± 4.01% for the six days. The percentage of remaining results requiring video display frame to interpret the movements of the female rats is within the range of 50% and 83%. Therefore, the mean of the percentage is 66.76 ± 5.35% for six days.

Accordingly, the aforementioned analysis indicates that as the number of days increases, the observer faces increasing difficulty when identifying the peak signals. Therefore, it shows that to identify the front and hind paws accurately, the observer needs the video display frame.

**Figure 9.**
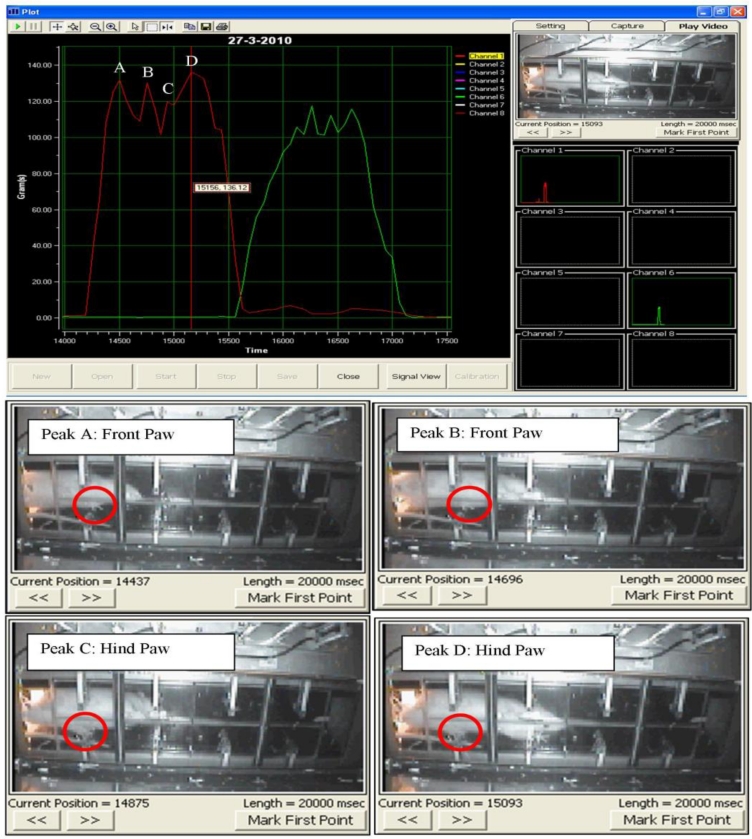
Uncertain occurrence of a peak signal. During the rat locomotion, the stepping forces were captured and displayed in real-time signal display frame and video display frame concurrently. The four peak signals which are showed in channel 1 are represented in **(A)** front paw **(B)** front paw **(C)** hind paw **(D)** hind paw.

Other researchers [10], have assumed that the first and second peaks represent the front and hind paws respectively. In fact, it is difficult to differentiate between the front and hind paw without the aid of the video display frame, as shown from the present study. Figure 9 clearly illustrates the uncertainty associated with the occurrence of peak signals. While Peak A represents the front paw, the second peak shown (Peak B) is also the front paw, whereas the third and fourth peaks (Peak C) and (Peak D) indicate the hind paws, respectively. From the results obtained, it has been proven that the assumption of Min *et al.* [10] is not realistically applicable for stepping force measurement. Hence, the use of the video display frame is essential for accurate observation.

Currently, using the existing devices, the researcher needs a great deal of time and patience to observe the motion of the rats. An observer needs to select an appropriate image that closely resembles the generated signal. When the rats are not willing to walk along the apparatus or remain in a resting state, he needs to wait patiently for the rat to walk voluntarily since some of the existing devices are not able to capture real-time motion. During the stage of data acquisition, the movement of the rat may not consistently produce two peaks that represent the front and hind paws as mentioned by Min *et al.* [10]. In order to obtain the required results, the rat might need to walk many times. Due to this factor, the stepping force of the rat will be affected. Therefore, the aforementioned phenomenon may produce both false negative and positive results.

## 4. Conclusions

Although the weight distribution model proposed by Min *et al.* [10] is considered a fair measurement to quantify the level of pain [10], our proposed apparatus has proven that the assumption of Min *et al.* [10] is not practically applicable for identifying the peak signal of the front paw and hind paw of the rodent model. The apparatus proposed is this paper includes a new feature, that is, it integrates a built-in infrared CCD camera integrated with the analgesimeter. Hence, the camera is able to capture the locomotion of the rats and synchronise the stepping force concurrently so that each step can be identified and interpreted correctly using the data acquisition system.

## Figures and Tables

**Table 1.** Precision and accuracy of each channel of analgesimeter.

**Channel**	**Precision**		**Accuracy (%)**
	**Intra-day**	**Inter-day**	

1	0.16 ± 0.20	0.66 ± 0.29	100.10 ± 0.20
2	0.12 ± 0.19	0.52 ± 0.27	100.01 ± 0.17
3	0.58 ± 0.86	0.73 ± 0.25	100.16 ± 0.45
4	0.08 ± 0.19	0.56 ± 0.56	99.66 ± 0.32
5	0.58 ± 1.37	0.70 ± 0.11	99.87 ± 0.13
6	0.34 ± 0.57	0.71 ± 0.34	100.36 ± 0.50
7	0.68 ± 0.09	0.88 ± 0.12	99.91 ± 0.18
8	0.35 ± 0.48	0.69 ± 0.23	99.42 ± 0.57

Note: Results are expressed as mean ± SD.
